# Complete genome of *Nanosynbacter* sp. strain BB002, isolated and cultivated from a site of periodontal disease

**DOI:** 10.1128/mra.00637-24

**Published:** 2024-11-27

**Authors:** Celine Grace F. Atkinson, Kristopher A. Kerns, Erik L. Hendrickson, Xuesong He, Batbileg Bor, Jeffrey S. McLean

**Affiliations:** 1 Department of Periodontics, University of Washington, Seattle, Washington, USA; 2 Department of Oral Health Sciences, University of Washington, Seattle, Washington, USA; 3 Department of Microbiology, ADA Forsyth Institute, Cambridge, Massachusetts, USA; 4 Department of Microbiology, University of Washington, Seattle, Washington, USA; Department of Biological Sciences, Wellesley College, Wellesley, Massachusetts, USA

**Keywords:** oral microbiome, microbial ecology, clinical microbiology, Saccharibacteria, Patescibacteria

## Abstract

*Nanosynbacter sp.* strain BB002, was isolated from the human oral cavity on its basibiont bacterial host *Actinomyces* sp. oral taxon 171 strain F0337, related to *Actinomyces oris*. As a member of the *Saccharibacteria* within the candidate phylum radiation group (CPR), its reduced genome facilitates the survival as an ultrasmall (<0.2 μm) epibiont.

## ANNOUNCEMENT

*Nanosynbacter lyticus* strain TM7x (accession CP007496.1) is a member of the Candidatus Saccharibacteria phylum (formerly TM7), and this type species is highly prevalent in the human oral cavity (1–3). As additional Saccharibacteria isolates were uncovered, defining traits for all Saccharibacteria include an ultrasmall cell size and reduced genome (2, 3). Saccharibacteria were later identified as members of the candidate phylum radiation (CPR), dominated by yet uncultivated groups, and are predicted to have a similar lifestyle since they all share reduced genomes (4, 5). The current GTDB taxonomy designation for the CPR is the superphylum Patescibacteria and Saccharibacteria is Class Saccharimonadia (6).

Here, we describe the complete genome sequence of strain BB002, which was previously isolated and characterized phenotypically (7). BB002 was isolated on a host bacterium *Actinomyces* sp. F0337, under IRB approval (ADA Forsyth: IRB #16-07) from the subgingival pocket of an individual with periodontitis (7). Briefly, periodontal samples were vortexed and filtered through 0.45 μm filters with a membrane (PVDF; Millipore, Burlington, MA) (7). Filtered ultrasmall cells were baited on multiple host bacteria, including F0337, in brain heart infusion (BHI) medium under microaerophilic conditions (<2% oxygen) at 37°C (7). Cultures were collected and passaged every 24 h for five passages and tested for Saccharibacteria via microscopy and PCR (7). BB002 was pulled down/baited on F0337 and was further cleaned by serial dilution and colony picking.

Genomic DNA was extracted using the Epicentre MasterPure kit (LGC Biosearch Technologies, FisherScientific, Berlin, DE) (1, 8, 9). Library preparation was executed in-house using the Nextera XT DNA Library Preparation Kit (Illumina, San Diego, CA) (10, 11). The library was sequenced on an Illumina Novaseq 6000 system (2 × 150 base pairs); default parameters were used for all software. SRA reads were controlled using BBDuk v35.14 (12). After QC, and reads were assembled using Unicycler v0.4.8 with the standard protocol (13). Additionally, the genome was annotated using the NCBI PGAP v6.6 using default parameters for bacterial annotation (14).

BB002 and its *Actinomyces* sp. host, F0377, were sequenced together with a combined total of 17,751,986 reads covering 2,662,797,900 bases (8). From this coverage, the BB002 complete genome was assembled into a single contig at 804,253 base pairs long and circularized by Unicycler v0.4.8 before being rotated to designate the dnaA gene as the start site. The average genome coverage was 84.0× with a G + C content of 47.87%. Gene annotation via NCBI PGAP v6.6 identified a total of 866 genes with 44 tRNAs for the BB002 genome. Among notable genes for BB002 include genes encoding for Type IV pili assembly, ATPase PilB, ABC-type antimicrobial peptide transport system (ATPase component), and Adenine-specific DNA methyltransferase.

Taxonomic classification of BB002 was examined using GTDB-Tk v2.3.2 (15). When compared to *N. featherlites* strain BB001, strain BB002 taxonomic classification was characterized by topology and ANI percentage (93%) resulting in a proposed name of *Nanosynbacter* sp. strain BB002. Additionally, HOMD 16S rRNA BLAST for BB002 displays 99.5% identity to *N. featherlites* BB001 (HMT-957) (CP040004.1).

## Data Availability

This genome has been deposited in GenBank under the accession number CP146064 and indexed under BioProject accession number PRJNA1074842. BB002 SRA details can be found under accession number SRR29249473.
